# A Rare Case of Severe Pelvic Organ Prolapse with Massive Perineal Hernia in a Nulliparous Woman: A Case Report and Literature Review

**DOI:** 10.3390/diagnostics15192481

**Published:** 2025-09-28

**Authors:** Andrea Rus, Andrei Manea, Andrei Cora, Béla Szabó, Ioana Hălmaciu

**Affiliations:** 1 Department of Radiology, County Emergency Clinical Hospital of Târgu Mureș, 54136 Târgu Mureș, Romania; demdeea@gmail.com (A.R.); cora.andrei@yahoo.com (A.C.); 2 Department of Obstetrics and Gynecology, County Emergency Clinical Hospital of Târgu Mureș, 54136 Târgu Mureș, Romania; 3 Doctoral School of Medicine and Pharmacy, George Emil Palade University of Medicine, Pharmacy, Science and Technology of Târgu Mureș, 540142 Târgu Mureș, Romania; 4 Department of Radiology, George Emil Palade University of Medicine, Pharmacy, Science and Technology of Târgu Mureș, 540142 Târgu Mureș, Romania; ioana.halmaciu@umfst.ro; 5 Department of Obstetrics and Gynecology, George Emil Palade University of Medicine, Pharmacy, Science and Technology of Târgu Mureș, 540142 Târgu Mureș, Romania; bela.szabo@umfst.ro

**Keywords:** grade IV pelvic organ prolapse, complex perineal hernia, early recurrence, rare pathology, absence of collagen disorders

## Abstract

**Background and Clinical Significance:** Advanced pelvic organ prolapse (POP) associated with perineal herniation of pelvic and abdominal organs is a sporadic occurrence in gynaecological practice. Generally, POP affects up to 50% of multiparous women at some point during their lives. Advanced forms (grade III or IV) represent less than 10% of all cases, with severe grade IV prolapse occurring in fewer than 2% of patients. **Case Presentation:** We report the case of a 48-year-old nulliparous woman with no prior surgical history and no known medical conditions at presentation. The patient presented with severe grade IV POP (Baden–Walker Classification), characterised by abdominal pain, vaginal bleeding and significant urinary incontinence. A computed tomography scan was performed, revealing an extremely large perineal hernia, containing the uterus, urinary bladder, and small bowel loops—a rare finding with only isolated cases reported in the medical literature. Surgical treatment involved a total intracapsular hysterectomy with right-sided adnexectomy and colpoperineorrhaphy. After the surgery, the overall status of the patient was good. However, less than two months later, she returned, complaining of a recurrence of the initial pathology, and was diagnosed with grade II/III POP recurrence despite having no connective tissue disorders or other classical predisposing factors such as pregnancies, pelvic surgery history or obstetric trauma. The case was further complicated by a femoral neck fracture, stage V chronic kidney disease, COVID-19 pneumonia, and a *Clostridium difficile* infection. All these complications led to the postponement of the gynaecological reintervention procedure. **Conclusions:** We emphasise the significant challenges in managing this kind of perineal hernia, under unusual conditions and without common risk factors. A personalised, multidisciplinary approach is required, including careful follow-up to prevent early recurrence.

## 1. Introduction

Pelvic organ prolapse (POP) is a relatively common condition among women, especially in the older population [1]. Studies have found a statistically significant association between advanced prolapse and classic risk factors identified in the literature [2], including advanced age, multiparity, elevated body mass index and foetal macrosomia [3]. Constipation has also been identified as a significant risk factor [4]. POP considerably impacts women’s quality of life, with 60.8% of affected women in rural areas of Pakistan reporting moderate to severe effects [5]. Prevalence rates vary, from 10.3% in rural Pakistan to as high as 40% globally [3]. However, a South Korean study [4] reported a much lower prevalence of 180 per 100,000 women over the age of 50 years.

Prolapse in the anterior compartment is frequent, primarily caused by vaginal birth trauma, connective tissue disorders, obesity and chronic intra-abdominal pressure increases, such as persistent cough or heavy physical exertion [6]. Patients often present with a feeling that “something is coming out” [7], describing a sensation of pressure in the vaginal area. Many accuse stress-induced urinary incontinence, difficulty emptying the bladder and urgency, all leading to recurrent urinary tract infections [8]. These symptoms negatively impact quality of life, causing discomfort, embarrassment, limitations in social activities and even painful sexual dysfunction [9]. Prolapse severity is evaluated using the Pelvic Organ Prolapse Quantification (POP-Q) system, allowing objective classification from stage 1 (mild prolapse) to stage 4 (complete prolapse) [10]. Anterior compartment prolapse is the most common type, accounting for 54% to 79% of cases requiring surgical intervention [11]. Treatment can be conservative, including pelvic floor muscle exercises and the use of pessaries, but if the symptoms persist or become worse and prolapse worsens, surgical intervention may be needed [6,12].

Over time, surgical techniques and management of these pathologies have differed. However, more surgeons are performing procedures such as anterior colporrhaphy and paravaginal repair, each coming with distinct advantages and disadvantages regarding recurrence rates and postoperative complications [13]. Other surgical approaches include sacrocolpopexy, which often has superior results to other vaginal procedures such as sacrospinous colpopexy or transvaginal mesh placements. However, every advantage is accompanied by a disadvantage; in this case, the benefits are counterbalanced by higher costs, longer operative times and slower recovery. Notably, when using the polypropylene mesh for anterior vaginal wall repair, the recurrence rate of the prolapse appears to be reduced. Still, risks such as urinary incontinence and mesh-related complications are increased, necessitating careful risk–benefit evaluations. With this in mind, no clear consensus exists on the best surgical method, with each technique having specific advantages and limitations [14]. A meticulous evaluation is crucial in choosing the optimal technique in each case, considering all factors such as patient age and prior surgeries.

## 2. Case Report

**Figure 1:**
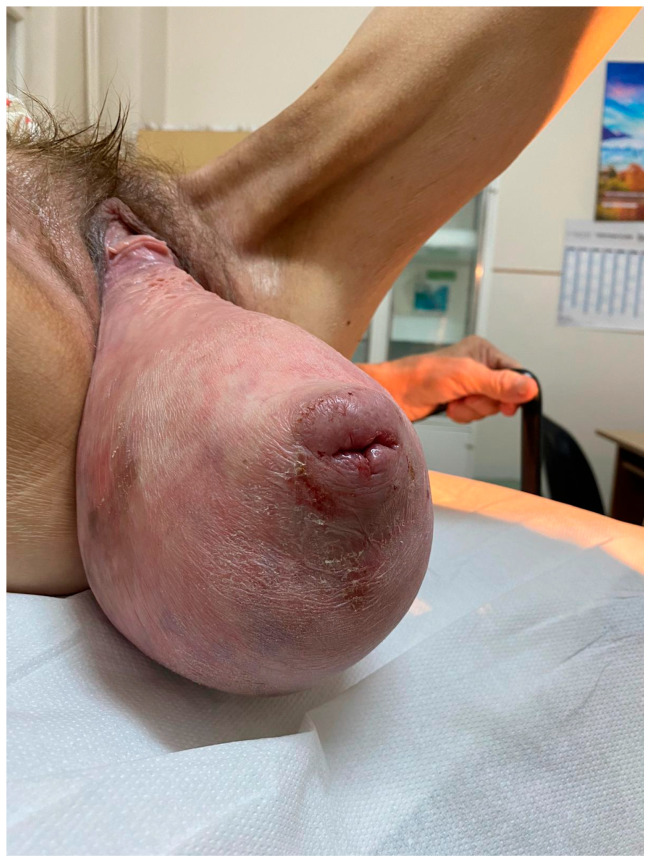
Upon direct visual inspection, the considerable size of the prolapse is evident, with the uterine cervix centrally located and the external cervical os appearing as a transverse slit without visible macroscopical pathological changes. Posteriorly, the hernia sac exhibits areas suggestive of tissue distress, including dehydration and early structural alterations that may progress to ulceration with continued exposure.

**Figure 2:**
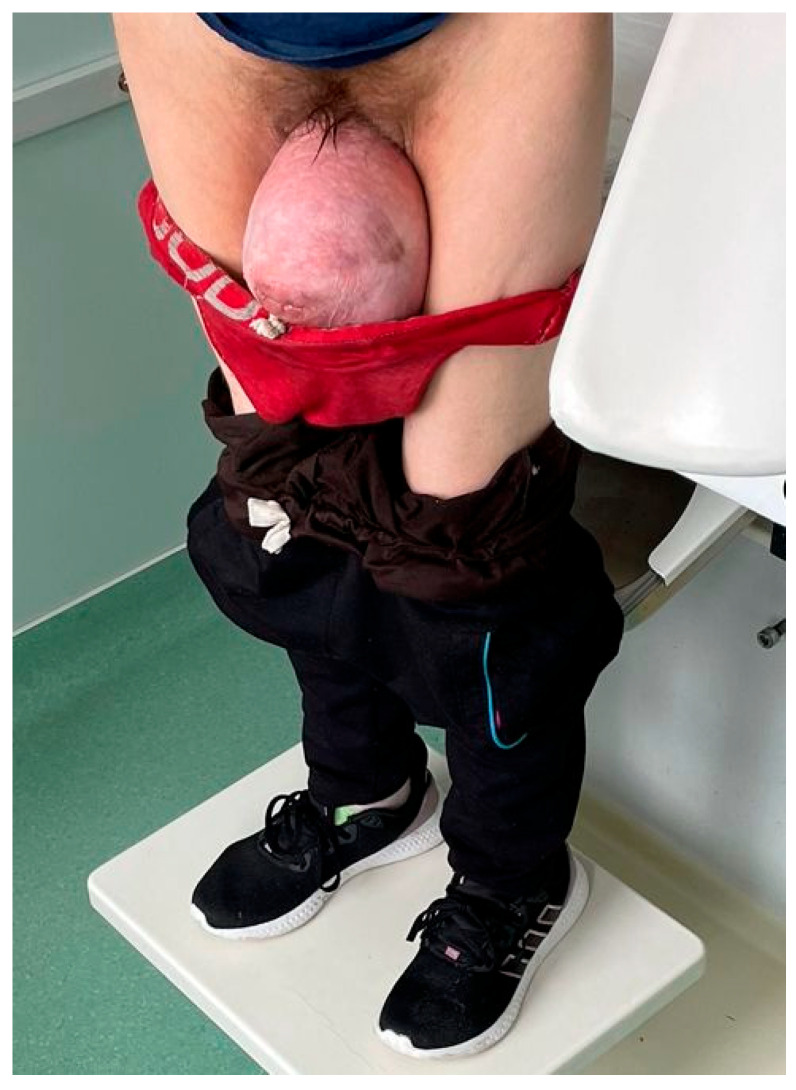
The image highlights the large perineal hernia, with a maximum diameter of 15 cm, with the patient in an upright position. Under gravity, the prolapse becomes more prominent and severe, exacerbating the symptoms and impairing postural comfort.

We report a rare case of a 48-year-old woman, declaratively without any pregnancy or surgical history and no known medical conditions at the time of the first medical presentation, apart from chronic kidney disease that was later discovered. She came to our medical unit complaining of abdominal pain, vaginal bleeding and significant urinary incontinence. Based on the clinical examination, the patient was diagnosed with severe grade IV POP, according to the Baden–Walker (Halfway) staging system [15,16]. The appearance of the patient at the initial presentation is demonstrated in Figure 1 and Figure 2.

**Figure 3:**
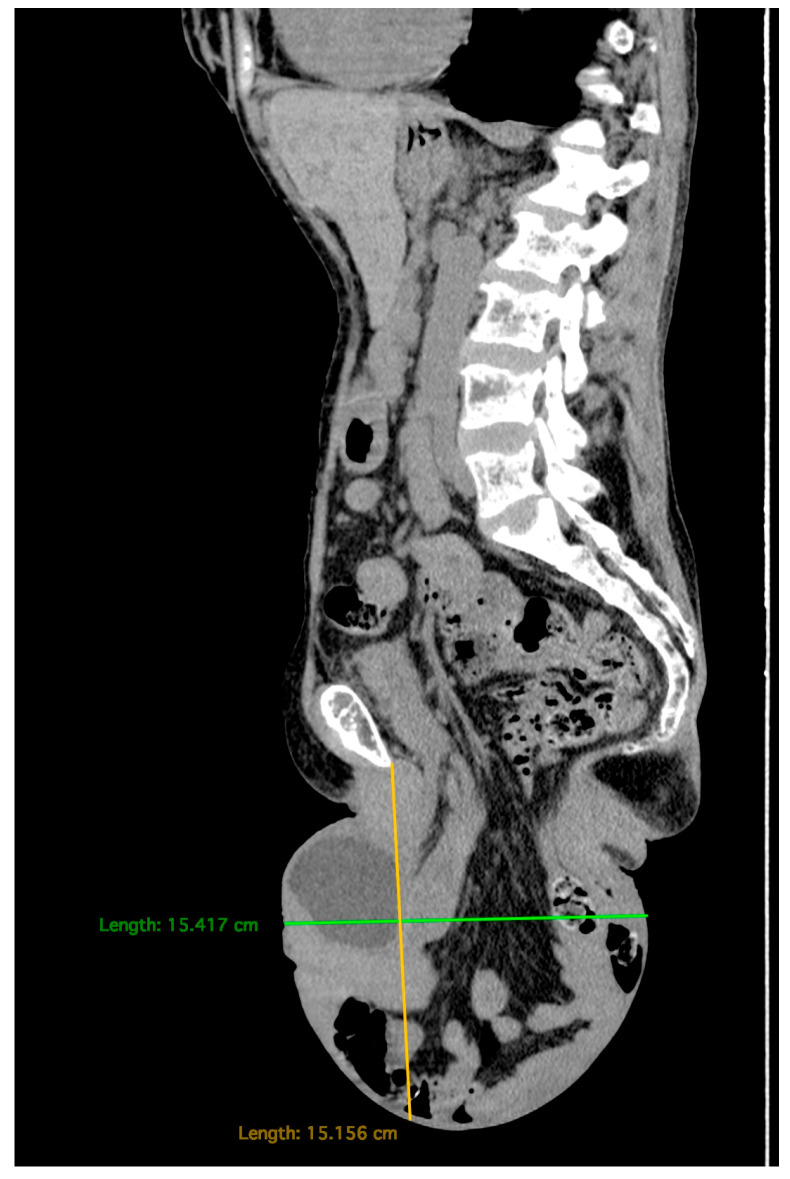
Native abdomen and pelvis, CT scan. Sagittal section. It shows the content and size of the hernia sac. The measured distance from the pubic symphysis to the lower pole of the hernia sac is 15.2 cm.

**Figure 4:**
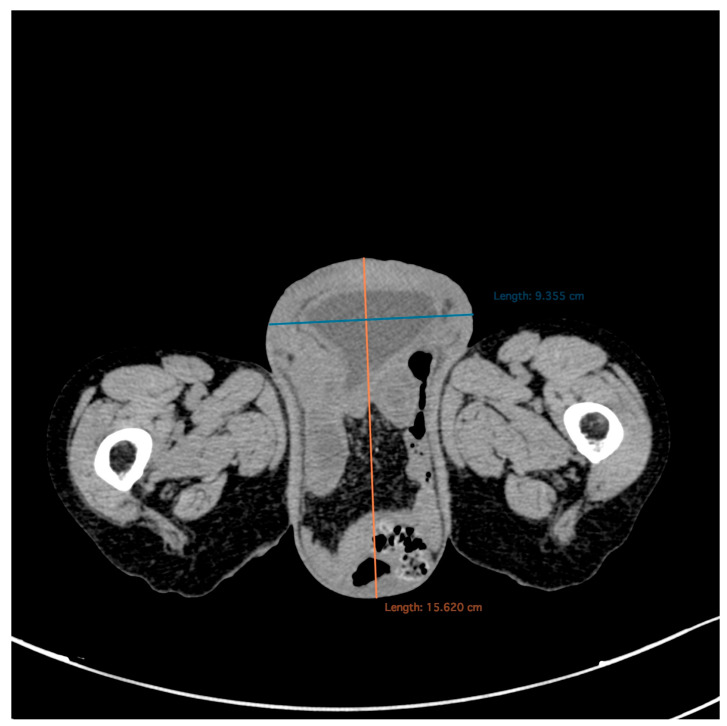
Native abdomen and pelvis, CT scan. Axial section. It shows the hernia sac with a diameter of 15.6/9.3 cm, with complete prolapse of the urinary bladder and the presence of bowel loops within the hernia sac.

Computed tomography (CT) scans of the abdomen and pelvis (Figure 3 and Figure 4) revealed a large perineal hernia containing the uterus, urinary bladder and small bowel loops—an extremely rare finding, with only isolated cases reported in the medical literature.

After preoperative preparation, on 6 December 2022, the patient underwent surgery under general anaesthesia. The procedure included total intracapsular hysterectomy with right adnexectomy, colpoperineorrhaphy and levator ani myorrhaphy. It was carried out laparotomically with a subumbilical approach. The detailed surgical technique involved mobilising the uterus above the umbilicus, skeletonising it, and resecting approximately 8–9 cm of the vaginal apex, followed by vaginal cuff closure and fixation to the round ligaments. Finally, the lead surgeon reconstructed the abdominal wall in anatomical layers, performing afterwards a high colpoperineorrhaphy.

Initially, postoperative care went well, and the patient recovered quickly. However, in less than two months, she returned, reporting the recurrence of the initial pathology. The patient was diagnosed with POP grade II/III recurrence, still without classical predisposing factors. The overall status of the patient noticeably declined, and after several multidisciplinary investigations, new diagnoses were made, though not related to the gynaecological problem: a fracture of the left femoral neck, the progression of chronic kidney disease to stage V, COVID-19 pneumonia with interstitial involvement and *Clostridium difficile* infection. On 2 February 2023, the patient was urgently readmitted, this time to the orthopaedic ward for treatment of the femoral neck fracture. She underwent right hip hemiarthroplasty with a cemented bipolar prosthesis. Given the new findings, multidisciplinary management was required to monitor renal function; administer antibiotics, anticoagulation and analgesia; and correct electrolyte imbalances. However, due to the patient’s unstable condition and comorbidities, reintervention with the gynaecological procedure was postponed until stabilisation of the more life-threatening issues.

In August 2023, after six months, the patient was again admitted to the haematology department, complaining of fatigue and pallor, and was diagnosed with moderate normochromic macrocytic anaemia. A CT scan was requested, revealing multiple osteolytic and osteosclerotic bone lesions, which raised a suspicion of multiple myeloma, subsequently ruled out by bone marrow histopathology examination. The lab results indicated hypercalcemia, azotaemia and elevated inflammatory markers. Following additional investigations, including a positive urine culture for *E. coli* and a clear chest X-ray, the patient was discharged with specific treatment recommendations and advised to return for follow-up evaluation within three months, advice that she ultimately did not follow.

## 3. Discussion

Recent specialised literature on patients who have undergone abdominoperineal resections for cancer indicates that recurrences after perineal hernia repair are remarkably common. A meta-analysis [17] including 172 patients evaluated recurrence rates after biological versus synthetic mesh repair, showing superior outcomes with synthetic mesh (29% recurrence) compared to biological mesh (39%). Moreover, combining mesh with a tissue flap further reduced recurrence rates to approximately 9%. We found a larger review [18] of 29 studies encompassing 347 procedures, which observed a recurrence rate of 22%, with no significant difference between the perineal and abdominal approaches. Another study [19] emphasised that women who underwent vaginal prolapse surgery with hysterectomy tended to have higher rates of both intraoperative and postoperative complications compared with those who had not had hysterectomy. This study review also highlighted a significant limitation: the lack of diversity in study populations, particularly in terms of ethnicity, age and comorbidities.

After we correctly diagnose uterine prolapse or perineal herniation, we face a new problem: the difference in the management of these conditions. Without a precise protocol for the management of these cases, we are bound to make mistakes that lead to an unsuccessful surgery or a higher recurrence rate in these patients. In a systematic review and narrative synthesis study [20] from 2023, the rates of healthcare-seeking behaviour varied widely, from 21.3% in Pakistan to 73.4% in California, USA. The included studies represented four distinct populations across six different countries. The authors concluded that the level of healthcare-seeking behaviour was generally low in low-income countries. Since all the data suggest that large perineal hernias carry a high recurrence rate, a combined approach (synthetic mesh plus flap) should be used to minimise this rate because it appears to be most effective.

Numerous studies in the literature have evaluated the effectiveness of various surgical techniques [21,22,23,24,25]. Pecorella et al. [26] published a comprehensive review of the literature and developed an algorithm for surgical decision-making for POP. The paper provides a synthesis of the techniques used in POP surgery, compares international guidelines and proposes an algorithm that simplifies the decision-making process. According to this synthesis, anterior colporrhaphy is recommended as the first-line option for the anterior compartment, posterior colporrhaphy remains standard for the posterior compartment, and an abdominal approach with sacrocolpopexy is the widely accepted option for apical prolapse. Furthermore, a conceptual transition from “sacrocolpopexy” to “sacropexy” is anticipated, allowing the surgeon to choose the anatomical portion that will be suspended from the promontory (cervix, cervical stump or vaginal stump). The technique selection is personalised according to the anatomy, patient preferences and comorbidities. The proposed algorithm helps clinicians choose the optimal surgical therapy for each patient, considering the severity of prolapse, preferences and anatomical aspects. By standardising the decision, the authors aim to improve outcomes and reduce the variability of interventions for POP.

The issue extends beyond merely aesthetic concerns, however. Most patients report various symptoms, with psychological involvement mainly left out. A 2018 paper by Laganà et al. [27] specifically addressed this issue, presenting a personal opinion rather than a clinical study. It discussed the impact of POP on women’s quality of life, with particular emphasis on its psychological and emotional dimensions. Women with this pathology often experience decreased self-esteem, depression, anxiety and problems in sexual relationships, leading to a general impairment of psychosocial well-being. The authors highlighted in their conclusions that the assessment of patients with uterine prolapse should also include psychological aspects, not just anatomical or functional ones. They recommended a multidisciplinary approach that combines surgical or medical treatment with psychological support and counselling.

Studies in the literature have reported that pelvic floor disorders, POP and stress urinary incontinence (SUI) harm sexual function and quality of life [27,28,29,30]. The main complaints are low libido, vaginal dryness, dyspareunia, discomfort, sexual dysfunction and emotional distress [28,29,31].

Validated questionnaires such as the Urogenital Distress Inventory-6 (UDI-6), Incontinence Impact Questionnaire-7 (IIQ-7), International Consultation on Incontinence Short Form (ICIQ-SF) and the Pelvic Organ Prolapse/Urinary Incontinence Sexual Function Questionnaire (PISQ-12) can assess the impact of these dysfunctions on quality of life [30]. In the context of acute presentation and multiple comorbidities, in the presented case, these questionnaires were not applied, but their importance is supported in the specialised literature. They provide a complete picture of how surgical treatment influences both physical and psychological well-being, underlining the importance of a multidisciplinary approach. Skorupska et al. [30] showed that a UDI-6 score > 33.33 correlated with an increased level of discomfort associated with urinary incontinence symptoms. Additionally, women with significantly impaired quality of life had scores > 9 on the IIQ-7 questionnaire [30].

Doğan et al. evaluated the effects of surgical treatment on sexual life among patients diagnosed with SUI and POP. All participants were assessed preoperatively and six months postoperatively. IIQ-7 and UDI-6 scores were significantly lower postoperatively, indicating a definite improvement in quality of life [31].

Hsieh et al. evaluated the benefits of laparoscopic sacrocolpopexy (LSCP) in POP, specifically analysing the incidence of de novo SUI and the impact of the surgical intervention on sexual function [32]. Although studies have demonstrated a significant decrease in PISQ-12 scores after the application of various surgical techniques in POP [33,34], Hsieh et al. reported a significant improvement in PISQ-12 scores after LSCP. LSCP has demonstrated high efficacy in the treatment of POP, with improved sexual function and reduced incidence of SUI, while maintaining a low rate of complications [32].

We align ourselves with the opinion of other studies, according to which the management of pathologies such as uterine prolapse or perineal hernia, beyond the anatomical, medical and aesthetic aspects, should also include psychological counselling before and after the procedure, given the proven emotional impact of these conditions. Thus, we emphasise again the need for a multidisciplinary approach, which also integrates the psychological treatment component.

Because our patient fell outside the usual postoperative or oncologic scenarios, these findings highlight the need to establish the best surgical strategy for each patient’s specific anatomy and individual risk factors. Moreover, they reinforce the importance of detailed imaging assessment and meticulous management to minimise the likelihood of recurrence and subsequent complications. Our patient had no history of pregnancy, had not undergone any prior surgeries and did not possess any typical risk factors for pelvic prolapse, such as obstetric trauma or connective tissue disorders [7]. A comprehensive review of the medical literature revealed very few cases similar to ours. One notable report described a 48-year-old nulliparous woman with grade IV POP accompanied by a large perineal hernia; remarkably, the patient was a virgin—an exceedingly rare feature in severe POP [20]. Comparing these two cases emphasises not only the rarity of such clinical presentations but also the value of a multidisciplinary evaluation and a personalised therapeutic plan when treating severe POP in the absence of traditional risk factors. In the few cases described, the patients have had a prior risk factor such as vaginal delivery, which is the primary cause of anterior compartment prolapse [6].

Table 1 summarises our case and similar cases involving nulliparous presentations from the literature, focusing on age, parity, organs involved, imaging, treatment, follow-up and recurrence.

Few studies in the literature have reported ureterohydronephrosis complicated by renal dysfunction as a complication of severe uterine prolapse. Thanasa et al. [42] presented a case report of a menopausal patient with three previous vaginal deliveries who developed bilateral ureterohydronephrosis and renal dysfunction in the context of severe uterine prolapse. In our case, the patient also had grade V renal failure, with uterine prolapse considered the main cause of the onset of renal dysfunction.

POP affects up to 50% of multiparous women during their lifetime. However, advanced stages (III or IV) represent fewer than 10% of cases, with stage IV occurring in less than 2% [6,11].

All of these data demonstrate that even severe POP can occur without the usual risk factors. Therefore, clinicians must conduct a rigorous clinical evaluation, including detailed imaging, rather than relying on statistical expectations [10,43]. Even if the initial surgery went well, the rapid recurrent state highlights the challenges in managing complex and atypical cases, like this one, and the importance of a coordinated, multidisciplinary approach, as well as the need for a close postoperative follow-up. Serious complications can arise in postoperative care. Our patient had hip fracture surgery, advanced renal disease and hospital-acquired infections, which further complicated her care, delayed reoperation and heightened overall risk, reinforcing again the need for comprehensive teamwork among specialists [9].

POP significantly affects women’s quality of life, both physically, by limiting daily activity, and psychologically, with decreased self-esteem, anxiety and depression. Age also plays an important role in accentuating physical limitations [44].

Numerous imaging methods have been proposed for screening, diagnosing, staging and monitoring POP, ranging from classical fluoroscopy to more advanced techniques such as CT, magnetic resonance imaging (MRI) and ultrasound [45]. These methods can be done statically or dynamically. A review article by Lipetsakia et al. from 2025 [45] evaluated all these imaging methods, concluding that no single method was superior to another. Most methods can diagnose POP moderately well in advanced stages, but further studies are required for diagnosing low-grade POP.

A 2023 study concluded that static MRI did not offer additional benefits over clinical evaluation of POP. However, dynamic studies can help detect abnormalities of the pelvic organs at rest and during contraction, straining and defecation [46].

Ultrasound studies are accessible and cost-effective and can be performed more quickly than other imaging methods. They provide a very accurate image of the pelvic floor and can be used for detecting POP in the anterior and middle compartments [47]. An article from 2025 by García-Mejido et al. described the first artificial intelligence-based method for evaluating POP, using videos of the trans-perineal ultrasound examination during the Valsalva manoeuvre [48]. The study showed an agreement of almost 100% with the expert examiner, making further studies combining artificial intelligence and ultrasound promising.

CT is not recommended for diagnosing POP due to the similarities in tissue density. However, in some situations, such as when patients cannot tolerate MRI, it can be the only option for imaging diagnosis [49]. Surgical planning can be done using CT images, evaluating the size and the complexity of the POP, as in our case. Due to the urgency of the case and the clinical confirmation of the diagnosis, we preferred to use a CT scan to gain a better perspective for the upcoming surgery.

Imaging studies are recommended in complex or recurrent POP cases. 2D/3D/4D transperineal ultrasound is a dynamic, accessible and practical examination. Still, it has limitations compared to dynamic pelvic MRI (dMRI) [50]. dMRI allows for simultaneous visualisation of the three pelvic compartments (anterior, apical, posterior) and the musculo-ligamentous structures, providing a complete assessment of the pelvic floor and facilitating the identification of dysfunctions with multicompartmental involvement [51]. dMRI guides preoperative planning and the choice of the optimal surgical strategy. According to the literature, it has superior performance to fluoroscopic defecography in multicompartmental pathology [52]. Welch et al. [53] presented a synthesis of the indications, interpretation and clinical applications of dMRI in POP in women, emphasising its preoperative utility in complex cases.

Another important aspect that emerges from the analysis of this case is the need for standardised management protocols for severe genital and perineal prolapse because the lack of clear guidelines determines a high variability in surgical outcomes and may contribute to high recurrence rates. At the same time, advanced imaging methods, such as CT and MRI, prove their value especially in atypical cases, where clinical examination or POP-Q classification do not capture the complexity of the anatomical situation. Additionally, comorbidities that develop along the way—such as chronic kidney disease, femoral neck fracture and nosocomial infections, as in our case—can directly influence prognosis and delayed reintervention, highlighting the fact that the management of these patients cannot be viewed exclusively from a gynaecological perspective. In this sense, non-gynaecological factors also become major prognostic elements, with an impact on survival and quality of life, again reinforcing the idea that these cases require a holistic assessment and rigorous interdisciplinary collaboration.

## 4. Conclusions

Our case is both rare and instructive for medical practitioners, helping clinicians recognise and manage severe POP in unusual contexts. The case highlights the importance of a thorough and careful evaluation to guide the selection of the most appropriate surgical approach, reminding us that every patient is unique and requires their own treatment plan. Even if the patient is relatively young, as in our case, advanced POP can still occur, even without the classical risk factors. Another key takeaway is the importance of imaging studies in correctly diagnosing and managing patients, as well as proper preoperative planning in managing such complex cases in multidisciplinary teams.

Although the initial surgical treatment was successful, complications can still arise afterwards. Each patient should be managed accordingly, with close collaboration among medical specialities. Long-term follow-up is always necessary, even when obvious risk factors remain absent.

Individualised planning is essential to minimise the risk of complications and recurrence. Moreover, a multidisciplinary team, involving gynaecology, urology, radiology and, when needed, psychological support, is crucial in achieving optimal outcomes for patients with severe or atypical POP.

## Figures and Tables

**Table 1:** Summary of the cases found in the literature involving large prolapse in nulliparous patients.

Author, Year	Age	Parity	Organs Involved	Imaging	Operation/ Treatment	Follow-Up Time/ Recurrence
Mahmoudnejad et al., 2024 [35]	65	0 (virgin)	Stage IV POP (procidentia)	-	Vaginal sacrospinous ligament fixation + partial vaginal resection	4 years/ No recurrence
Ai et al., 2018 [36]	29	0 (virgin)	Stage II cystocele, stage III uterine prolapse, and stage II rectocele	CT	Conservative (Gellhorn pessary)	3 months/ No recurrence
Song et al., 2024 [37]	48	0	Cervical leiomyoma and stage III uterine prolapse	-	Robot-assisted vaginal hysterectomy followed by sacrocolpopexy	3 months/ No recurrence
Payá Ten et al., 2021 [38]	52	0 (virgin)	Stage IV POP (procidentia)	CT	Colpocleisis	Not mentioned/ No recurrence
Ko & Lo, 2011 [39]	21	0	Stage IV POP-Q	-	Sacrospinous ligament hysteropexy, anterior and posterior vaginal wall repair and perineorrhaphy	3 months/ No recurrence
Rana et al., 2022 [40]	17	0 (virgin)	Stage III POP (uterus)	-	Not done	-
Nigam et al., 2012 [41]	21	0	Cervix protrusion (apparent stage III POP secondary to large Nabothian cyst)	Ultrasound	Cyst excision	6 weeks/ No recurrence

## Data Availability

Data available on request from the authors. The data are not publicly available due to privacy.

## Abbreviations

The following abbreviations are used in this manuscript:

POP	Pelvic organ prolapse
POP-Q	Pelvic Organ Prolapse Quantification
CT	Computed tomography
MRI	Magnetic resonance imaging
dMRI	Dynamic magnetic resonance imaging
UDI-6	Urogenital Distress Inventory-6
IIQ-7	Incontinence Impact Questionnaire-7
ICIQ-SF	The International Consultation on Incontinence Short Form
SUI	Stress urinary incontinence
PISQ-12	Pelvic Organ Prolapse/Urinary Incontinence Sexual Function Questionnaire
LSCP	Laparoscopic sacrocolpopexy
